# Optimization of Liver Decellularization Maintains Extracellular Matrix Micro-Architecture and Composition Predisposing to Effective Cell Seeding

**DOI:** 10.1371/journal.pone.0155324

**Published:** 2016-05-09

**Authors:** Panagiotis Maghsoudlou, Fanourios Georgiades, Holly Smith, Anna Milan, Panicos Shangaris, Luca Urbani, Stavros P. Loukogeorgakis, Benedetta Lombardi, Giuseppe Mazza, Charlotte Hagen, Neil J. Sebire, Mark Turmaine, Simon Eaton, Alessandro Olivo, Jasminka Godovac-Zimmermann, Massimo Pinzani, Paul Gissen, Paolo De Coppi

**Affiliations:** 1 Department of Stem Cells and Regenerative Medicine, UCL Institute of Child Health and Great Ormond Street Hospital, 30 Guilford Street, London, WC1N 1EH, United Kingdom; 2 MRC Laboratory for Molecular Cell Biology, University College London, London, WC1E 6BT, United Kingdom; 3 London Center for Nephrology, University College London, London, WC1N 1EH, United Kingdom; 4 Institute for Liver and Digestive Health, University College London, London, WC1N 1EH, United Kingdom; 5 Department of Medical Physics & Bioengineering, University College London, London, WC1N 1EH, United Kingdom; 6 Department of Histopathology, UCL Institute of Child Health and Great Ormond Street Hospital, London, WC1N 1EH, United Kingdom; 7 Division of Bioscience, University College London, London, WC1N 1EH, United Kingdom; Michigan Technological University, UNITED STATES

## Abstract

Hepatic tissue engineering using decellularized scaffolds is a potential therapeutic alternative to conventional transplantation. However, scaffolds are usually obtained using decellularization protocols that destroy the extracellular matrix (ECM) and hamper clinical translation. We aim to develop a decellularization technique that reliably maintains hepatic microarchitecture and ECM components. Isolated rat livers were decellularized by detergent-enzymatic technique with (EDTA-DET) or without EDTA (DET). Histology, DNA quantification and proteomics confirmed decellularization with further DNA reduction with the addition of EDTA. Quantification, histology, immunostaining, and proteomics demonstrated preservation of extracellular matrix components in both scaffolds with a higher amount of collagen and glycosaminoglycans in the EDTA-DET scaffold. Scanning electron microscopy and X-ray phase contrast imaging showed microarchitecture preservation, with EDTA-DET scaffolds more tightly packed. DET scaffold seeding with a hepatocellular cell line demonstrated complete repopulation in 14 days, with cells proliferating at that time. Decellularization using DET preserves microarchitecture and extracellular matrix components whilst allowing for cell growth for up to 14 days. Addition of EDTA creates a denser, more compact matrix. Transplantation of the scaffolds and scaling up of the methodology are the next steps for successful hepatic tissue engineering.

## Introduction

Decellularized tissues have provided an option for engineering tissue both for transplantation and for disease modeling. However, ideal scaffolds should have architectural and mechanical characteristics allowing migration and proliferation of introduced cells, a defined biodegradation profile, and a minimal immune response [1,2]. For complex organs, such as the liver, scaffold choice is limited to decellularized materials, wherein cell removal from the whole-organ yields a three-dimensional extracellular matrix (ECM) [1,3].

Rat liver decellularization was initially performed using increasing concentrations of sodium dodecyl sulphate (SDS), followed by Triton-X 100 (TX100) [4]. This was succeeded by methods using increasing concentrations of TX100, followed by SDS [5], a combination of trypsin, EDTA and TX100 [6], and a combination of TX100 and ammonium hydroxide [7]. The inclusion of ion-chelating agents, such as EDTA and EGTA, was derived from their routine use for hepatocyte isolation. The general methodology based on detergents such as TX100 and SDS has been duplicated in many laboratories with slight variations [8–12]. Decellularization based on SDS and TX100 has also been scaled-up to larger animals [13–16]. However, current decellularization protocols cause substantial harm to the ECM and may render the vasculature too porous for successful transplantation. This is effectively shown in vascular casting images in rat livers decellularized by 1% SDS and 1% TX100 that demonstrate destruction of the blood vessel network [9].

We have previously decellularized intestine, lung and esophagus [17–20] using deionized water (dH_2_O), a low concentration of a mild detergent (sodium deoxycholate; SDC) and an enzyme to remove DNA remnants. This detergent enzymatic treatment (DET) [21] preserves scaffold microarchitecture and the microvascular network and has allowed successful clinical transplantation of human tracheas [22].

The aim of this study was to develop a decellularization protocol for rat liver that will preserve microarchitecture and ECM components. We aim to examine the interplay between the scaffold structural proteins and the DET and EDTA chemicals so as to create a scaffold that will allow long-term transplantation.

## Materials and Methods

### Harvest of organs

This study was carried out in accordance with the recommendations in the Animal (Scientific Procedures) Act 1986. The Home Office approved the study protocol (licence number 70/2716). 250–300 g Sprague-Dawley rats (n = 100) were sacrificed by CO_2_ inhalation. The abdomen was sterilized with 70% ethanol and a U-shaped incision was performed to expose the abdominopelvic cavity. The abdominal inferior vena cava (IVC) and portal vein (PV) were identified and the PV was cannulated with a 24G cannula (BD, UK), which was secured in place with a 3–0 silk suture (Ethicon, UK). The abdominal IVC was ligated using silk sutures proximal to the right renal vein and the IVC was sectioned. The diaphragm was used as a holding point to release the whole liver from the supporting tissue. The whole procedure was carried out with special caution not to damage the Glisson’s capsule, which surrounds the organ.

### Decellularization

For DET treatment, the PV was connected to a peristaltic pump (Masterflex, UK) and perfused with dH_2_O (18.2 mΩ/cm) for 36 hours at 4°C. For the EDTA-DET treatment, rat livers were perfused with 2mM EDTA (Sigma, UK) for 15 minutes followed by dH_2_O for 36 hours at 4°C. Both DET and EDTA-DET rat livers were then transferred at room temperature and perfused with 4% SDC (Sigma, UK) for 6 hours followed by perfusion of 500 kU/ml DNase-I (Sigma, UK) in 1M sodium chloride (NaCl; Sigma, UK) for 3 hours. Flow rate was 4.5 ml/min (dH_2_O) and 6.5 ml/min (SDC and DNase).

### Histology

Samples were fixed in 4% paraformaldehyde (PFA; Sigma, UK), dehydrated in graded alcohol, paraffin embedded and sectioned at 5μm. Tissue slides were stained with haematoxylin and eosin (H&E; Leica, Germany), Masson’s Trichrome (MT; Leica, Raymond A Lamb, BDH Chemicals Ltd), Picrosirius Red (PR; Polysciences Inc., Germany), Elastin Van Gieson (EVG; VWR, Leica, Raymond A Lamb), and Alcian Blue (AB; BDH Chemicals Ltd, Cellpath Ltd) stains. Appropriate positive controls were used to ensure histological stains were performed correctly (small intestine for MT, PR and AB, skin for EVG). For all histological samples three biological and technical replicates were assessed using standard H&E prior to any special stains to ensure homogeneity. All images shown are representative samples.

### Immunohistochemistry

Following rehydration of the paraffin-embedded slides, and quenching of endogenous peroxidase activity using 1% H_2_O_2_ (Sigma, UK), antigen retrieval was performed using pepsin (Sigma, UK) at a concentration of 1 mg/ml in 1N HCl (Sigma, UK), at 37°C for 60 minutes. Slides were then permeabilized with 0.2% Triton X-100 (Sigma, UK) for 60 minutes and blocked in 1% BSA (Sigma, UK) for 30 minutes. Primary antibodies were used against fibronectin (Abcam, UK), laminin (Abcam, UK), collagen I (Abcam, UK), collagen III (Abcam, UK), and collagen IV (Abcam, UK) at dilutions of 1:100, in BSA 1% overnight. Staining was visualized using the ImmPRESS (Vector Laboratories, CA, USA) detection system. Once diaminobenzidine (DAB substrate kit, BD Pharmingen) staining was developed, sections were dehydrated and mounted in Eukitt mounting medium (Fluka, Sigma, UK). Appropriate fresh tissue and no primary antibody controls were used to ensure appropriate tissue positivity. Three biological and technical replicates were assessed. All images shown are representative samples.

### DNA quantification

DNA was isolated using a tissue DNA isolation kit (PureLink Genomic DNA MiniKit, Invitrogen, UK) following the manufacturer’s instructions. Briefly, the samples were digested overnight using Proteinase K and a digestion buffer. DNA samples were purified using alcohol washes and measured spectrophotometrically (Nanodrop, Thermo Scientific, US). Optical densities at 260nm and 280 nm were used to estimate the purity and yield of nucleic acids. For all quantifications at least three biological and technical replicates were obtained.

### Collagen quantification

The collagen content of native and decellularized tissue was quantified using the total collagen assay kit (QuickZyme Biosciences, The Netherlands). Briefly for collagen, the samples were hydrolyzed in 6N HCl at 95°C for 20 hours, the hydrolysates were mixed with a chromogen solution staining hydroxyproline residues and color was developed at 60°C for 1 hour. The absorbance for each sample was determined at 555 nm using a Infinite 200 Pro microplate reader (Tecan, US) and the collagen quantity was calculated from a standard curve from known concentrations of pure collagen hydrolysates. For all quantifications at least three biological and technical replicates were obtained.

### Elastin quantification

The elastin content of native and decellularized tissue was quantified using the FASTIN elastin assay (Biocolor, UK) according to the manufacturer’s instructions. Briefly, the samples were homogenized, and elastin was solubilized in 0.25 M oxalic acid. Two consecutive incubations were performed at 95°C to ensure complete extraction of elastin. Extracts were incubated with 5,10,15,20-tetraphenyl-21H,23H-porphine tetrasulfonate (TPPS) dye, and absorbance was determined at 555 nm spectrophotometrically (Tecan Infinity, US). Elastin concentrations from a standard curve were used to calculate the elastin content of the tissue. For all quantifications at least three biological and technical replicates were obtained.

### Glycosaminoglycan (GAG) quantification

The GAG content of native and decellularized tissue was quantified using the Blyscan GAG Assay Kit (Biocolor, UK). Briefly, the tissues were digested with papain at 65°C for 18 hours and aliquots of each sample were mixed with 1,9-dimethyl-methylene blue dye and reagents from the GAG assay kit. The absorbance at 656 nm was measured spectrophotometrically (Tecan Infinity, US) and compared to standards made from bovine tracheal chondroitin-4-sulfate to determine the GAG content. For all quantifications at least three biological and technical replicates were obtained.

### Scanning Electron Microscopy (SEM) and quantification

Samples were fixed in 2.5% glutaraldehyde in 0.1 M phosphate buffer (PBS; Sigma, UK) and left for 24 hours at 4°C. Following washing with 0.1 M PBS, they were cut into segments of approximately 1 cm length and cryoprotected in 25% sucrose (Sigma, UK), 10% glycerol in 0.05 M PBS (pH 7.4) for 2 hours, then fast frozen in Nitrogen slush and fractured at approximately -160°C. The samples were then placed back into the cryoprotectant at room temperature and allowed to thaw. After washing in 0.1 M PBS (pH 7.4), the material was fixed in 1% OsO_4_ / 0.1 M PBS (pH 7.3) at 3°C for 1½ hours and washed again in 0.1 M PBS (pH 7.4). After rinsing with dH_2_O, specimens were dehydrated in a graded ethanol-water series to 100% ethanol, critical point dried using CO_2_ and finally mounted on aluminium stubs using sticky carbon taps. The material was mounted to present the fractured surfaces across the parenchyma to the beam and coated with a thin layer of Au/Pd (approximately 2nm thick) using a Gatan ion beam coater. Images were recorded with a 7401 FEG scanning electron microscope (Jeol, US). For quantification purposes a blinded observer assessed images taken at 200x using the measure tool in ImageJ (NIH, US). For all samples examined with SEM three biological and technical replicates were assessed. Prior to SEM analysis tissue homogeneity was assessed using standard histology.

### Synchrotron-based x-ray phase contrast imaging (XPCI)

Measurements were performed at the biomedical beamline (I17) of the European Synchrotron Radiation Facility in Grenoble, France. The samples were placed approximately 150 m from the source (a 21-pole, 15 cm period wiggler), to ensure spatially coherent illumination. The beam was monochromatized by a fixed-exit Laue/Laue silicon (111) double crystal to 26 keV (ΔE/E~0.02%) and filtered using 0.8 mm of copper and 3 mm of aluminium. The sample had been placed on a PI miCos rotation stage (PI mi-Cos GmbH, Eschbach, Germany) to enable tomographic acquisitions. Images were recorded by a FReLoN CCD camera coupled to a 47 μm thick Gd_3_Ga_5_O_12_scintillator. The effective pixel size was 3.5x3.5 micron^2^. 2000 projections over 360° were acquired, with an angular step of 0.18°. Images were phase-retrieved using the “single-distance” method developed by Paganinet al, and 3D reconstructions performed using standard filtered back-projections [23]. For all samples examined with XPCI two biological and technical replicates were assessed. Prior to XPCI analysis tissue homogeneity was assessed using standard histology.

### Vascular network imaging

Vascular network imaging was carried out on scaffolds as described previously [18]. For visualization of the vascular tree, 1% Trypan Blue (Sigma, UK) was perfused into the scaffold at a rate of 2 ml/min. An iPhone 4S (Apple, US) was used to film the infusion and iMovie to separate still shots. The movies were uploaded on Fiji (NIH, US), the borders of the scaffolds delineated, and following a threshold placement for the white background, intensity and area covered by the infused dye measured across the stillshots. For all vascular network imaging at least three biological and technical replicates were obtained. Analyses shown in the figures are representative samples.

### Protein extraction, separation and in-gel protein digestion

A proof-of-concept proteomics analysis was performed comparing fresh liver, DET and EDTA-DET scaffolds (n = 1). Prior to proteomics analysis a punch biopsy and standard histology confirmed appropriate decellularization. Proteins extracted from fresh liver samples, EDTA-DET and DET treated liver samples were separated by 12% SDS-PAGE, under reducing conditions. Proteins were visualized by silver staining (ProteoSilver Plus, Sigma, UK), and bands (32 horizontal slices for each sample) excised from the gel lane and destained using a solution containing 100 mM sodium thiosulphate and 30 mM potassium ferricyanide in ratio 1:1. Samples were reduced by 10 mM DTT and alkylated with 100 mM iodoacetamide using the ProGest Investigator Instrument (DigiLab, Genomics Solutions, Cambs, UK) according to the established protocol [24]. Finally, each dry gel piece was rehydrated in 30 μL of 50 mM ammonium bicarbonate solution containing 250 ng of Trypsin Gold, Mass Spectrometry grade (Promega, Madison, USA), and incubated at 37°C overnight. The trypsinolysis was stopped with 0.1% formic acid (FA), and tryptic peptides were eluted, vacuum dried, and dissolved in 0.1% FA for LC−MS/MS.

### Mass spectrometry

LC−MS/MS analysis was performed with an LTQ-Velos mass spectrometer (Thermo Fisher Scientific, UK). Peptide samples were loaded using a Nanoacquity UPLC (Waters, UK) with Symmetry C18 180 μm × 20 mm (Waters part number 186006527) trapping column for desalting and then introduced into the MS via a fused silica capillary column (100 μm i.d.; 360 μm o.d.; 15 cm length; 5 μm C18 particles, NikkyoTechnos CO, Tokyo, Japan) and a nanoelectrospray ion source at a flow rate at 0.42 μL/min. The mobile phase comprised H_2_O with 0.1% FA (buffer A) and 100% acetonitrile with 0.1% FA (buffer B). The gradient ranged from 1% to 30% buffer B in 95 min followed by 30% to 60% B in 15 min and a step gradient to 80% B for 5 min with a flow of 0.42 μL/min. The full scan precursor MS spectra (400−1600 m/z) were acquired in the Velos-Orbitrap analyzer with a resolution of r = 60 000. This was followed by data dependent MS/MS fragmentation in centroid mode of the most intense ion from the survey scan using collision induced dissociation (CID) in the linear ion trap: normalized collision energy 35%; activation Q 0.25; electrospray voltage 1.4 kV; capillary temperature 200°C; and isolation width 2.00. The targeted ions were dynamically excluded for 30 s, and this MS/MS scan event was repeated for the top 20 peaks in the MS survey scan. Singly charged ions were excluded from the MS/MS analysis, and XCalibur software version 2.0.7 (Thermo Fisher Scientific, UK) was used for data acquisition.

### Protein identification

96 Raw MS files (32 for each experimental condition tested) were loaded and assembled by MaxQuant (version 1.4.1.2) and visualized by Perseus (version 1.4.1.3) software platform. The peak list generated by Quant.exe (the first part of MaxQuant) was searched using the Andromeda search engine against rat FASTA files (RAT.fasta.gz) downloaded from the UNIPROT Web site: ftp://ftp.uniprot.org/pub/databases/uniprot/current_release/knowledgebase/proteomes, last modified 19/2/2014. Selected parameters for Max-Quant analysis included the trypsin enzyme specificity, 2 missed tryptic cleavages, oxidation of methionine and acetylation of protein N-terminal as variable modifications and cysteine carbamidomethylation as fixed modification. In addition, the following parameters were applied to perform the database search: a minimum peptide length of 6 amino acids, a minimum of 1 peptide identified, a minimum of 1 razor + unique peptide and minimum 1 of unique peptide, top 6 MS/MS peaks per 100 Da, a peptide mass tolerance of 10 ppm for precursor ions, and a tolerance of 0.5 Da for MS/MS peaks. All proteins were filtered according to a false discovery rate (FDR) of 0.01% applied at both peptide and protein levels and a maximum peptide posterior error probability (PEP) of 1. MaxQuant output files were subsequently uploaded into Perseus in order to visualize and combine in one matrix the results obtained for the 3 different experimental conditions as well as to add GO term association for each protein group and to obtain data for the Venn Diagram.

### Cell culture and scaffold seeding

HepG2 cells were cultured using DMEM/F12 (Life Technologies, UK) supplemented with 10% FCS (Sigma, UK) and trypsinized and counted prior to seeding. DET liver scaffolds (n = 3) were divided in lobes (n = 15) and placed in individual petri dishes with high glucose DMEM and 1% penicillin/streptomycin (Sigma, UK). Cells were resuspended in 1*10^6^/ml concentration and 2*10^6^ injected per lobe multifocally (~30 injections/lobe) using a 33G needle (TSK laboratory, Japan). Seeded scaffolds were maintained in hepatocyte medium in a humidified environment at 37°C with 5% CO_2_ for up to 14 days. Medium was changed every 2 days. At days 1, 4 and 14 following seeding, the scaffolds were placed in 4% PFA and assessed by histology and immunofluorescence.

### Immunofluorescence

Seeded scaffolds were fixed in 4% PFA overnight, paraffin embedded and sectioned at 5μm. Sections were rehydrated, permeabilized (0.2% TX100, 30 min) and heat-mediated antigen retrieval was performed using a citrate buffer. Primary antibodies were used against Ki67 (Abcam, UK) and cleaved-caspase 3 (Cell Signalling, UK) (dilution 1:100) overnight at 4°C. Sections were incubated with Alexa-488 conjugated secondary antibody (Life Technologies) (dilution 1:100) at room temperature for 1 hour. Nuclei were counterstained with DAPI (dilution 1:1000) and Sudan Black was used to quench auto fluorescence. For quantification, a minimum of 500 cells over 8–10 fields of view at x63 magnification were counted for each time point in a blinded experiment. For all quantifications three biological and technical replicates were assessed.

## Results

**Fig 1:**
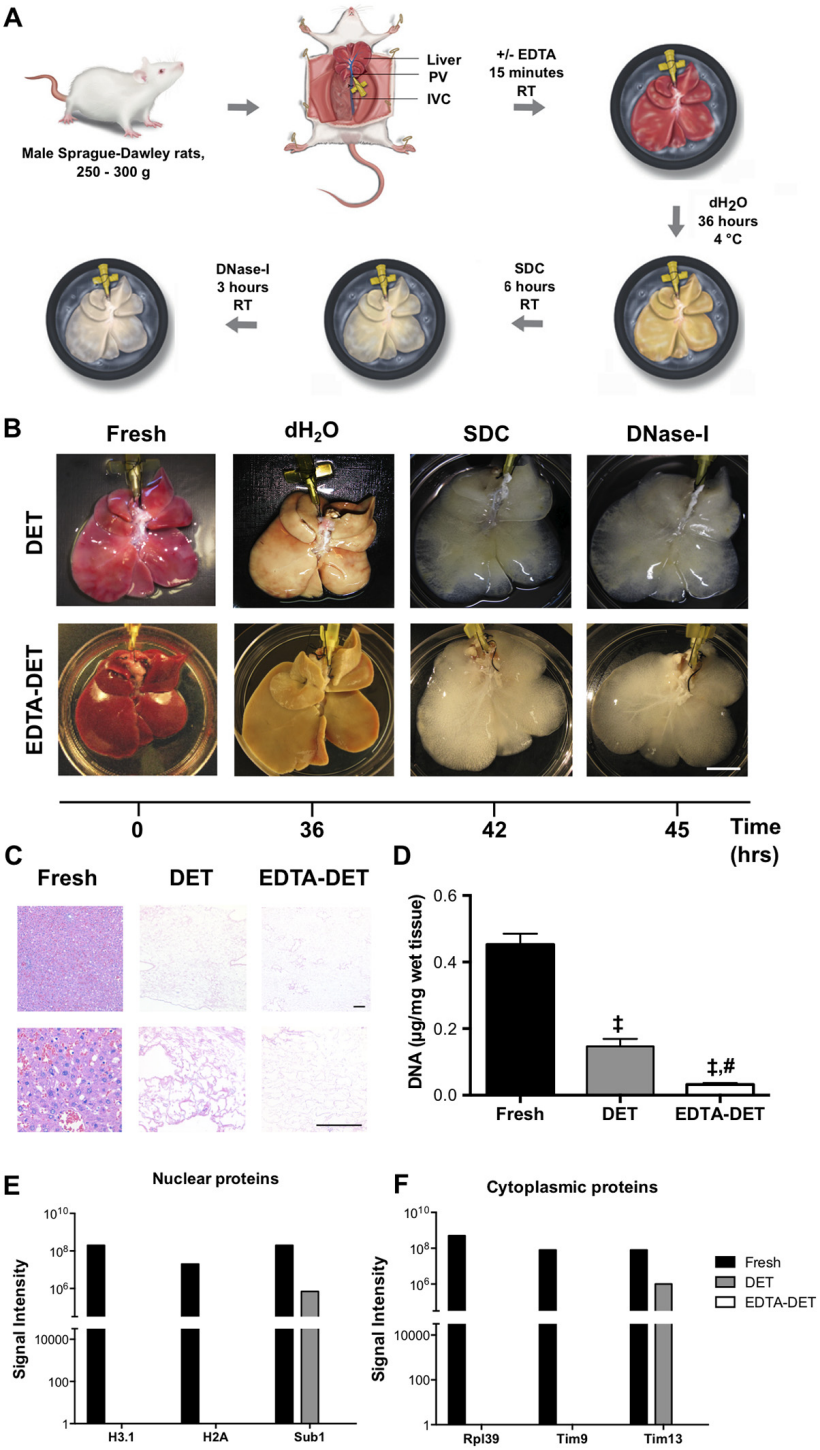
Decellularization of the rat liver is achieved following one cycle of DET and EDTA-DET treatments. (A) Timeframe and infusion of solutions for the DET and EDTA-DET protocols. (B) Macroscopic appearance of the liver scaffolds showed no difference between the DET and EDTA-DET scaffolds. Following dH_2_O addition, the livers became blanched, with SDC and DNase addition resulting in the livers becoming transparent. (C) H&E staining demonstrated absence of cells in sections from DET and EDTA-DET scaffolds. (D) DNA quantification reduced DNA in DET and EDTA-DET scaffolds compared with fresh tissue (p<0.001). (E-F) Histograms showing the signal intensities, associated to relevant nuclear proteins (E) and cytoplasmic proteins (F); ‡: p<0.001, compared to fresh tissue, #: p<0.001, compared to DET scaffold, scale bar in macroscopic images: 2cm, scale bar on histology: 100μm.

Following the first step of the DET (dH_2_O), the livers acquired a blanched appearance. Treatment with SDC and DNase led to translucency with vessels visible internally. There were no macroscopic differences observed between the rat livers pre-treated with EDTA and those treated with DET only (Fig 1A and 1B). Absence of cells was observed in sections stained with H&E (Fig 1C), while DNA quantification revealed a significant reduction in DNA amount between the fresh liver and liver subjected to either decellularization treatment (p<0.001; Fig 1D). Addition of EDTA further reduced the DNA amount compared to the DET treatment (p<0.001). Quantitative shotgun proteomics confirmed decellularization showing an absence of selected nuclear (Ntf2, H2A) and cytoplasmic (Rpl39, Tim9) markers from both EDTA-DET and DET only treated liver samples (Fig 1E and 1F). Moreover, EDTA-DET treatment was more efficient in removing cellular proteins as the nuclear protein Sub1 and the cytoplasmic protein Tim13 could be identified in the DET sample (Fig 1E and 1F).

**Fig 2:**
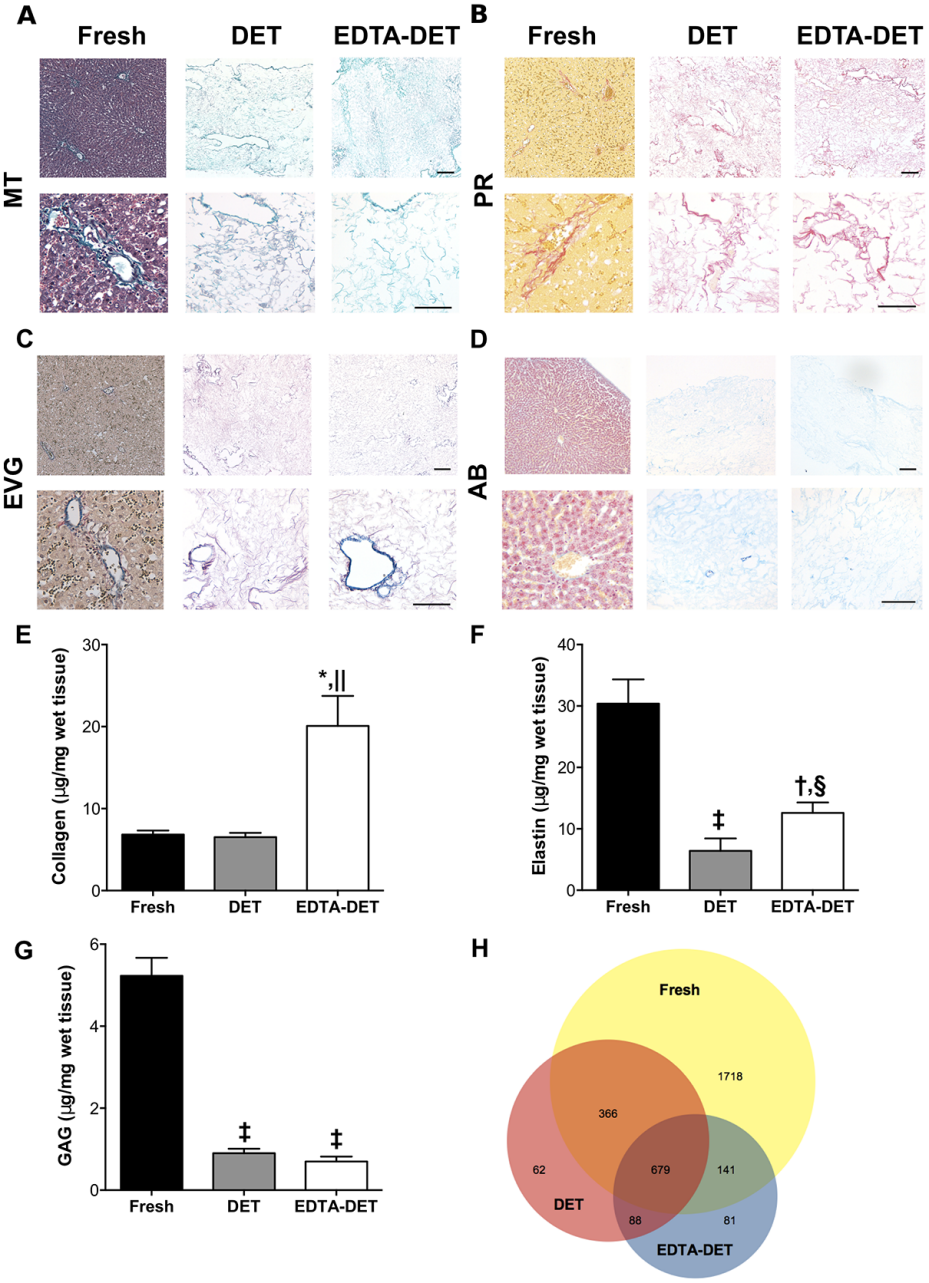
Decellularization preserves ECM components, with a higher amount of collagen and elastin present in the EDTA-DET scaffold. (A) MT staining demonstrated acellularity in both scaffolds, showing composition only by connective tissue. (B) PR staining demonstrated composition mostly by collagen fibers. (C) EVG staining revealed maintenance of elastin fibers in the inner surface of blood vessels. (D) AB staining confirmed the presence of GAG in both scaffolds. (E) Collagen was significantly increased in EDTA-DET scaffolds (p<0.01) compared with fresh tissue. (F) Elastin significantly decreased in DET (p<0.001) and EDTA-DET (p<0.01) scaffolds compared with fresh tissue. Elastin was higher in EDTA-DET scaffolds compared with DET scaffolds (p<0.05). (G) GAG quantification showed that while both DET and EDTA-DET scaffolds had significantly reduced GAG amount, EDTA-DET scaffolds had significantly less when compared to DET scaffolds (p<0.001). (H) Venn Diagram showing the number and the distribution over sample types of proteins identified in Fresh, DET and EDTA-DET treated liver tissues; *: p<0.05, †: p<0.01, ‡: p<0.001, compared to fresh tissue, §: p<0.05, ||: p<0.01, compared to DET scaffold, scale bar: 100μm.

**Fig 3:**
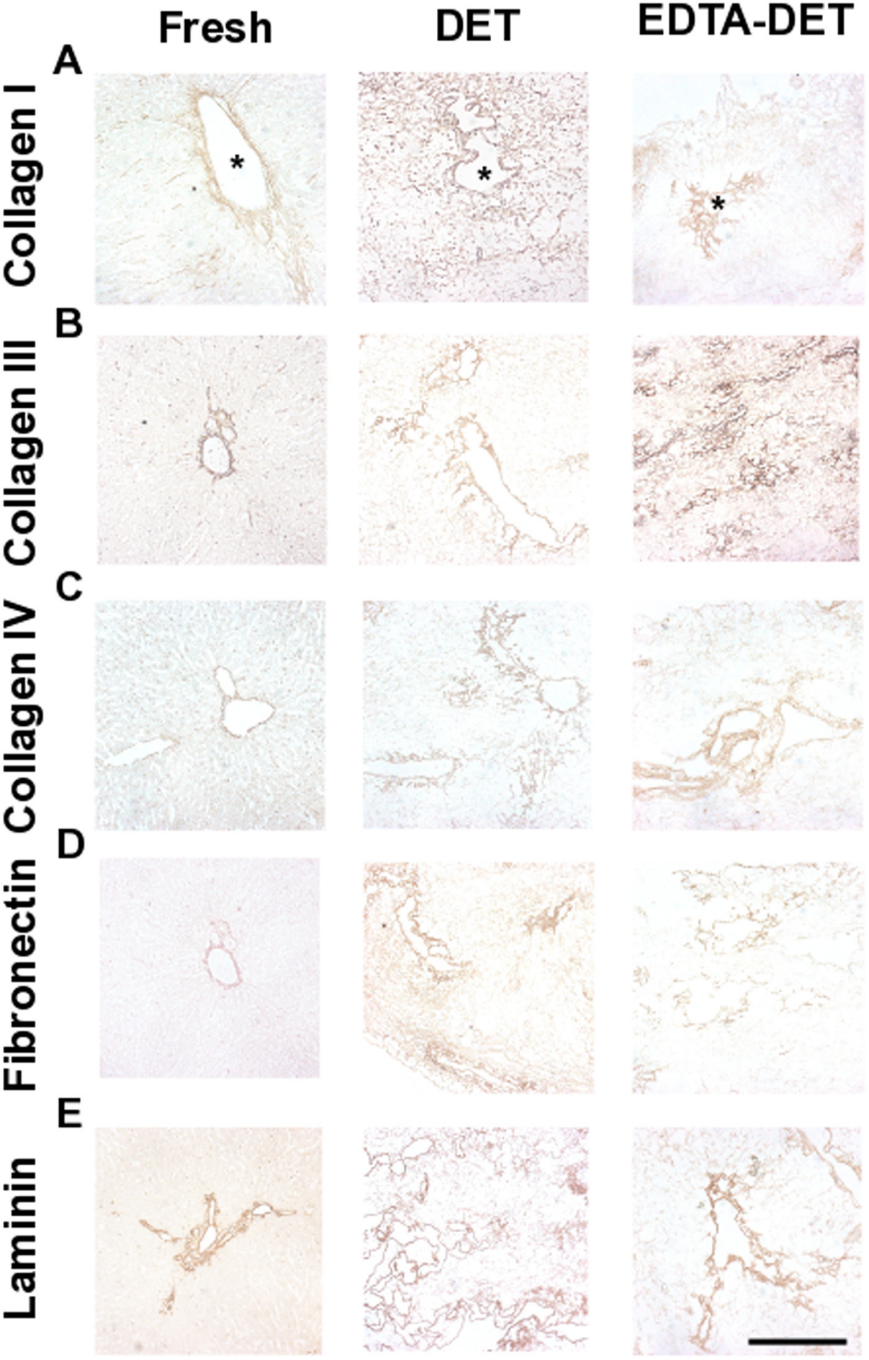
Immunostaining demonstrates the preservation of ECM proteins in the decellularized scaffolds. (A) Collagen I staining in fresh tissue was positive as fine strands in the parenchymal space as well as around the blood vessels (asterisk). This was preserved following decellularization with a strong signal from vascular structures both in DET and EDTA-DET scaffolds. (B, C) Collagen III and collagen IV staining demonstrated a similar distribution and preservation in fresh tissue and decellularized scaffolds. Weak parenchymal staining was observed, and a strong signal surrounding vascular and biliary structures. EDTA-DET scaffolds demonstrated slightly increased staining in the parenchymal space when compared to DET scaffolds. (D) Fibronectin showed strong staining around the main blood vessels in fresh tissue with a more distributed signal pattern in decellularized scaffolds. (E) Laminin showed strong staining around the main blood vessels in fresh tissue with a more distributed signal pattern in decellularized scaffolds; scale bar: 100 μm.

**Fig 4:**
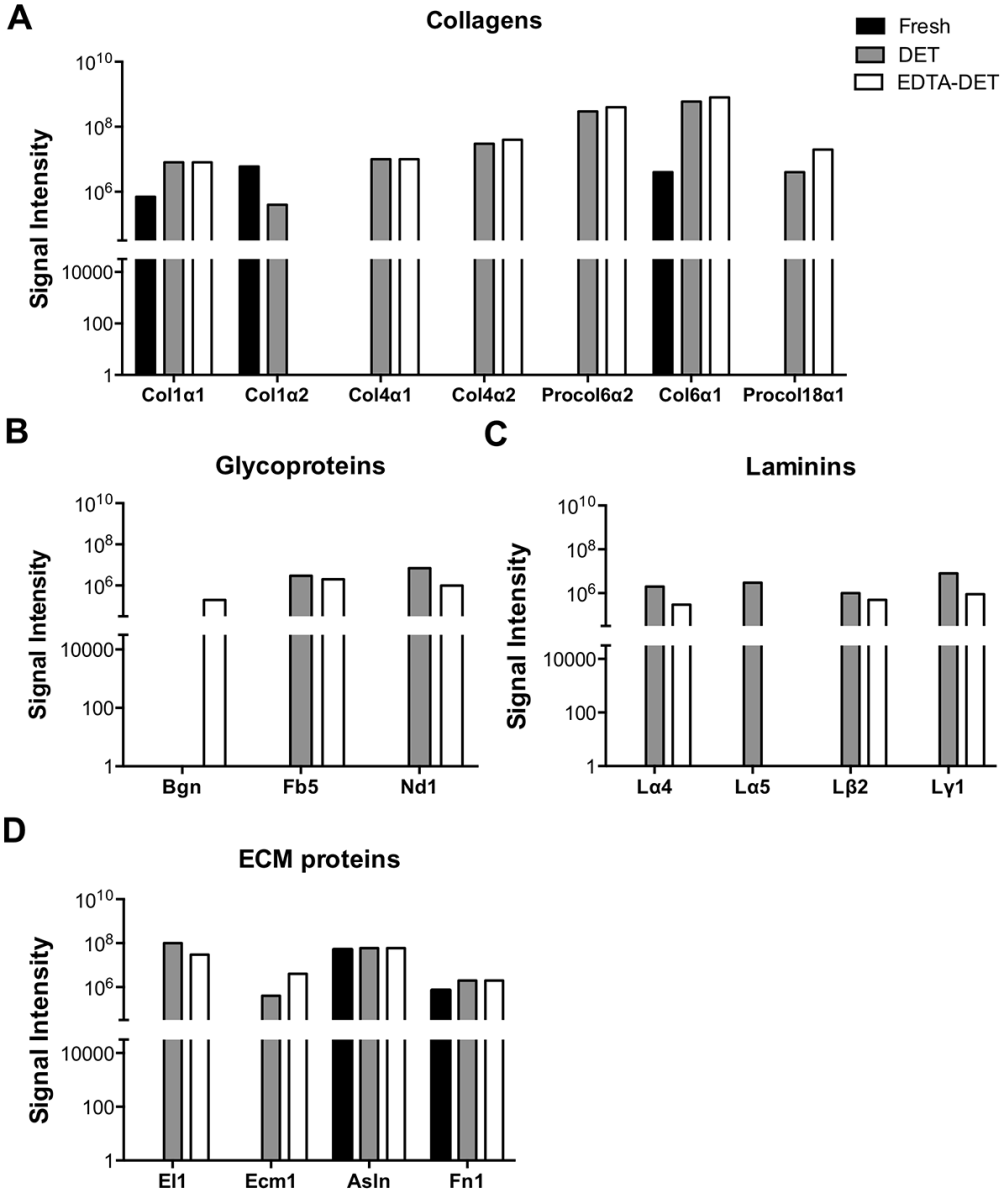
Proteomics demonstrate the preservation of ECM components in the decellularized scaffolds. Histograms showing the signal intensities, based on the summed extracted ions counts, measured for each peptide identified and associated to proteins belonging, accordingly to the GOCC classification, to the class of Collagens (A), Glycoproteins (B), Laminins (C) and Extracellular Matrix Proteins (D).

ECM components were assessed by means of staining and quantification. MT staining demonstrated that the cellular component was detectable only in the fresh tissue (Fig 2A). PR staining showed that the connective tissue was mostly composed of collagen fibers and was more compact closer to blood vessels in both fresh and decellularized tissue (Fig 2B). EVG staining confirmed maintenance of elastin fibers in the inner surface of blood vessels in both fresh and decellularized livers (Fig 2C). AB staining confirmed the presence of GAG in the fibers of both DET and EDTA-DET scaffolds (Fig 2D). Collagen quantification of the DET scaffolds demonstrated no significant changes when compared to fresh tissue (Fig 2E). EDTA-DET scaffolds had increased collagen compared to both fresh tissue (p<0.05) and DET scaffolds (p<0.01; Fig 2E). Elastin quantification demonstrated a decrease in both DET (p<0.001) and EDTA-DET (p<0.01) scaffolds to approximately 20–40% of fresh tissue (Fig 2F). In similar fashion to collagen, the elastin content of the EDTA-DET scaffolds was higher than DET scaffolds (p<0.05; Fig 2F). GAG content of both DET and DET-EDTA scaffolds was significantly reduced compared with fresh tissue (p<0.001; Fig 2G); however, there was no difference in content between DET and DET-EDTA scaffolds. Immunostaining demonstrated collagen I in fresh liver as fine strands in the parenchymal space, as well as around the blood vessels (Fig 3A). This was preserved following decellularization with a strong signal from vascular structures both in DET and EDTA-DET scaffolds. Immunostaining against collagen III and IV demonstrated a similar distribution in both fresh and decellularized tissues (Fig 3B and 3C). Weak parenchymal staining was observed, with a strong signal surrounding vascular and biliary structures. Strong staining of fibronectin was observed around the main blood vessels in fresh tissue with a more distributed signal pattern in decellularized scaffolds (Fig 3D). Immunostaining against laminin was positive only around major blood vessels in both fresh and decellularized tissues (Fig 3E). Proteomic analysis revealed 679 proteins in common amongst fresh tissue, DET and EDTA-DET scaffolds, while 231 proteins were uniquely identified in the decellularized scaffolds (Fig 2H). The main ECM constituents were identified in the decellularized samples and could not be detected in fresh tissue. For example, proteins belonging to the collagen family (IV, VIα2 and XVIII), glycoproteins such as fibulin (Fb5), nidogen (Nd1) and the laminin family of glycoproteins were detected in DET and EDTA-DET scaffolds, but were undetectable in the fresh sample (Fig 4A–4C). The same trend was observed in the ECM proteins (Fig 4D). A full list of identified proteins with the indication of the intensity measured in each sample is summarized in the supplementary information (S1 Table).

**Fig 5:**
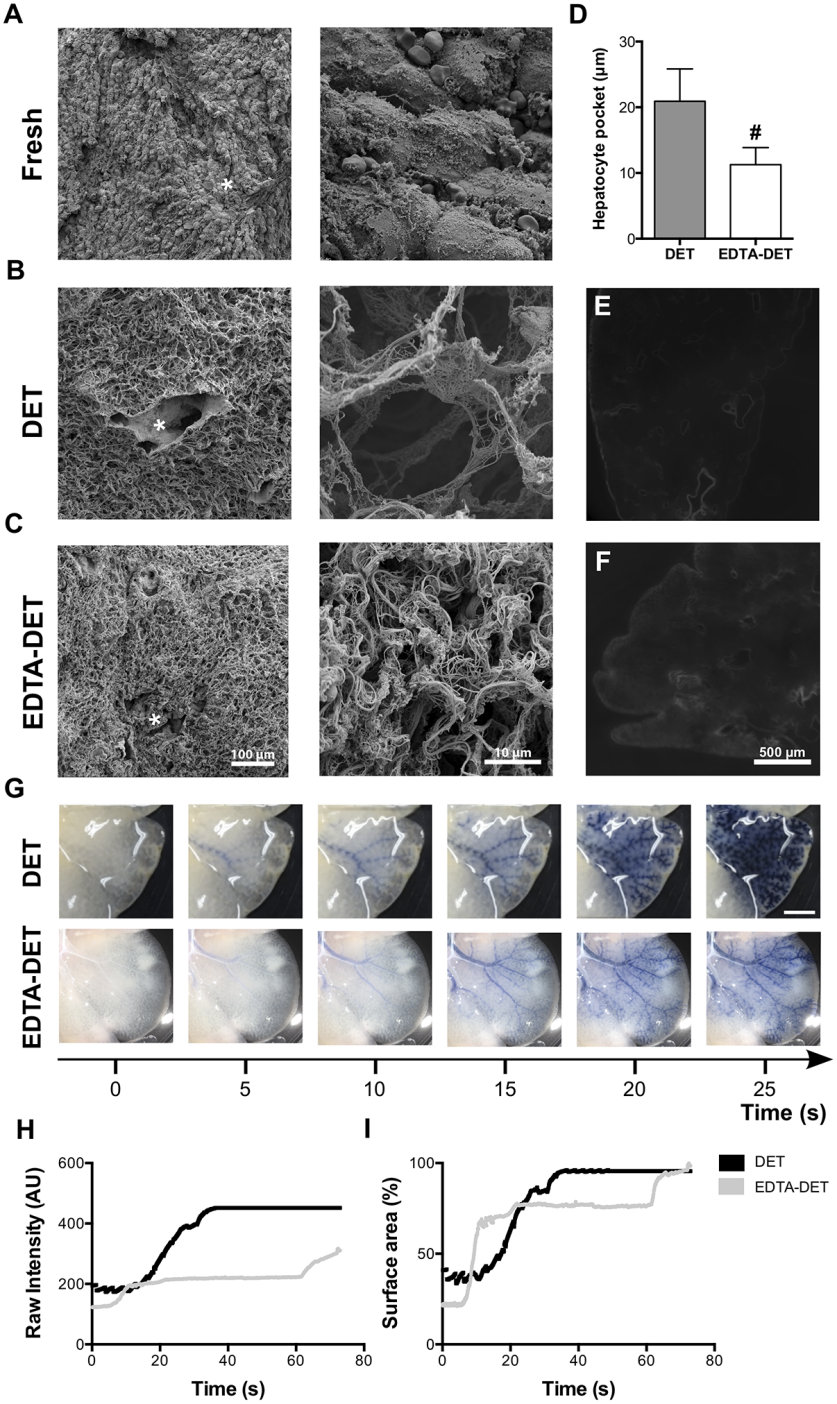
Addition of EDTA to the protocol makes the matrix more compact in the parenchyma and vasculature. (A-C) SEM confirmed acellularity in the scaffolds, demonstrating composition by a three-dimensional network of connective tissue fibers arranged in a honeycomb-like manner. The structure of the portal triads was identified clearly at low magnification (asterisk). (C) Scaffolds prepared with EDTA-DET were more tightly packed compared to the DET scaffolds, as the porous structure appeared to be compressed. (D) Quantification of the hepatocyte pockets in the EDTA-DET scaffold showed a reduction to approximately 50% of the size in the DET scaffolds. (E, F) Synchrotron-based x-ray phase contrast imaging corroborated these results, demonstrating a denser scaffold in scaffolds pre-treated with EDTA. (G) Infusion of trypan blue dye via the portal vein showed maintenance of the vascular network architecture. There was no dye leakage through the walls to the surrounding tissue; the dye followed the regular pathway of flow to the IVC. Macroscopically, the DET scaffolds were seen to possess a denser vascular network with the dye diffusing through the vessels more readily. (H) Dye intensity within the DET scaffolds followed an S-shaped curve, reaching a maximum point at approximately 35 seconds. Dye intensity within the EDTA-DET scaffold increased in a less steep S-shaped manner with the exponential part lasting 45 seconds instead of 15. Maximum intensity was reached at 75 seconds. (I) These results were paralleled by the quantification of surface area of dye distribution with the point of maximal surface area coverage having a difference of 40 seconds between the two scaffolds; #: p<0.001, compared to DET scaffold, scale bar: 1cm.

SEM further confirmed the complete absence of cells, while the scaffolds consisted of a three-dimensional network of connective tissue fibers arranged in a honeycomb-like structure. The portal triad structure was identified clearly at low magnification in fresh, DET and EDTA-DET livers (Fig 5A–5C, asterisk). Interestingly, scaffolds prepared with EDTA-DET were more tightly packed compared to the DET scaffolds, as the porous structure appeared to be compressed (Fig 5C). Quantification confirmed the change in microarchitecture, demonstrating a reduction of the hepatocyte pocket from 20.9±0.5 μm to 11.3±0.3 μm (p<0.001; Fig 5D). Synchrotron-based XPCI also showed denser tissue in the EDTA-DET scaffolds compared to DET (Fig 5E and 5F). While the major vessels can clearly be seen in both scaffolds, the more compact nature of the tissue makes the shade of grey in the image of the EDTA-DET scaffold overall more intense, which, in a phase-retrieved image, correlates directly with increased density (S1 and S2 Videos).

Trypan blue perfusion through the PV demonstrated maintenance of the vascular network with no leakage of the dye to the surrounding tissue, as the dye flowed to the IVC (Fig 5G, S3 and S4 Videos). Macroscopically, the DET scaffolds seemed to possess a more compact vascular network with the dye diffusing through the vessels more readily (Fig 5G). Quantitative analysis of the dye distribution using Fiji (NIH, USA, 1.47h) confirmed this. Intensity of the dye infusion through the DET scaffolds followed an S-shaped curve, reaching a maximum point at approximately 35 seconds (Fig 5H). Dye intensity within the EDTA-DET scaffold increased in a less steep S-shaped manner with the exponential part lasting 45 seconds instead of 15 (Fig 5H). Maximum intensity was reached at 75 seconds. These results were paralleled by the quantification of surface area of dye distribution with the point of maximal surface area coverage having a difference of 40 seconds between the two scaffolds (Fig 5I). This is in keeping with the data from SEM and XPCI showing a more compact appearance of the tissue following EDTA-DET treatment.

**Fig 6:**
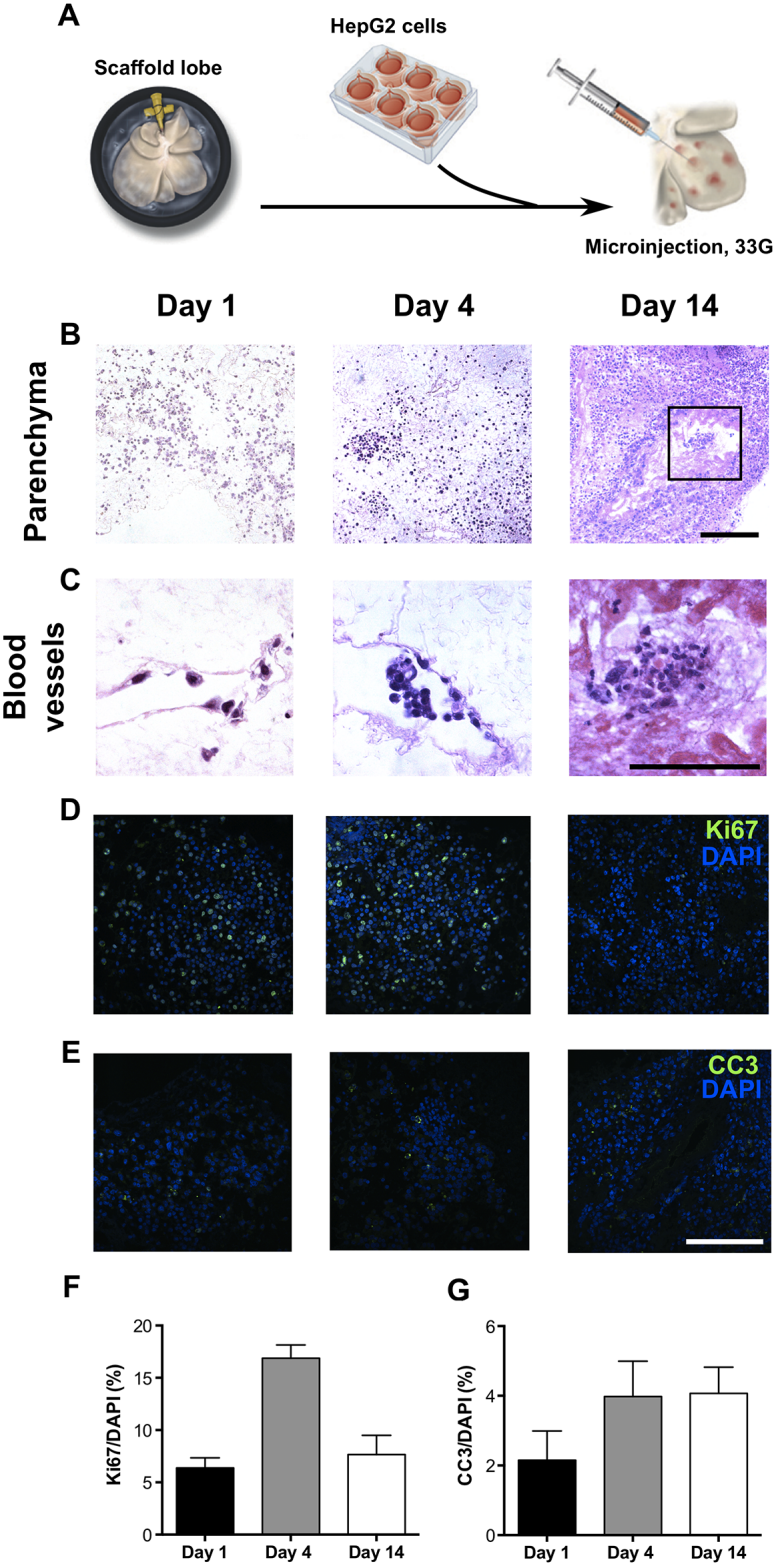
Seeding of the DET scaffolds by microinjection demonstrates complete repopulation at 14 days. (A) DET scaffolds were microinjected with HepG2 cells in a multifocal manner and the seeded constructs harvested at day 1, 4 and 14. (B) H&E staining demonstrated a wide distribution across the scaffold at day 1 and a complete repopulation at 14 days (B). At higher magnification, cells were seen repopulating the hepatic spaces, forming colonies and secreting their own ECM at 14 days (C, asterisk). Ki67 staining demonstrated proliferation at days 1 and 4, with only a few ki67-positive cells at 14 days (D). Quantification of ki67-positive cells as a percentage to DAPI-positive cells (F). Staining for CC-3 showed a few positive cells across the three time-points (E), with quantification demonstrating a non-significant increase (G); scale bar: 100μm.

Having identified the DET scaffolds as a more suitable option for tissue engineering due to better preservation of micro-architecture and ECM components, the scaffold biocompatibility was assessed with seeding of a hepatocellular cell line. HepG2 cells were microinjected in a multifocal manner in the DET scaffolds and seeded constructs were harvested at day 1, 4 and 14 (Fig 6A). H&E staining demonstrated a wide distribution of cellular engraftment across the scaffold at day 1 and complete repopulation at 2 weeks (Fig 6B). At higher magnification, histology showed the cells repopulating the empty hepatic spaces and forming colonies (Fig 6C). Immunofluorescence staining against ki67 demonstrated high numbers of proliferating cells at the first two time-points and only a few ki67-positive cells at 14 days (Fig 6D). Quantification by a blinded observer showed 6.4±1%, 16.9±1.3% and 7.7±1.8% of ki67 positive cells at days 1, 4 and 14 respectively (Fig 6F). Staining for CC-3 demonstrated a few apoptotic cells across the three time-points (Fig 6E). Quantification of the CC-3-positive cells showed a non-significant increase from 2.2±0.8% to 4±1% to 4.1±0.8% at days 1, 4 and 14 (Fig 6G).

## Discussion

Hepatic tissue engineering has so far had limited success in animal transplantation studies, in part due to the vascular leakiness of the decellularized scaffolds. We describe a rat hepatic scaffold obtained using a gentle decellularization process that preserves microarchitecture, ECM components, and maintains a suitable environment for growth of reseeded cells.

We have firstly described an effective DET decellularization in pig trachea [25] and, in addition to our successful clinical application of DET-decellularized trachea in a child [22], we have more recently explored this simple treatment in more complex organs such as the lung and the intestine [17–19]. The DET uses mild decellularization agents at low concentrations, however, due to the compact and highly cellular nature of hepatic tissue, more challenges could potentially be present and therefore additional treatments may be necessary. We thus compared decellularization using our normal DET agents versus a protocol in which EDTA was also used.

The decellularization protocol was slightly modified when compared to our work in the trachea, esophagus, intestine, and lung. We used longer perfusion steps of dH_2_O and SDC, the latter being determined by the time required to reach transparency. In order to keep the liver fully inflated during perfusion, the perfusion speed was increased from 4.5 ml/min (in the dH_2_O step) to 6.5 ml/min (in the SDC and DNase-I steps), with the complete protocol lasting 45 hours. Protocols published so far have used a variety of perfusion speeds with protocols lasting from 2 to 52 hours [4–16,26,27]. The main factors of a decellularization protocol that determine its duration are the chemicals used, their concentration and the flow rate. Decellularization with preservation of microarchitecture should involve mild chemicals, at low concentrations and correspondingly low flow rates. Shorter protocols (2–3 hours) either had high perfusion rates(10–30 ml/min), high concentrations of SDS and TX100, or both [5,8,11,12]. At 45 hours we achieved decellularization using a flow rate of 5–6.5 ml/min. This admittedly may be difficult for all species, however, we have recently demonstrated for the first time that complete decellularization of whole human liver with a preserved architecture still requires both SDS and TX100 [26]. The need for gentler decellularization methodologies that avoid SDS and TX100 led to our comparison of the DET and EDTA-DET protocols. In rat liver, addition of EDTA further reduced the DNA content compared with DET alone. The concentration of EDTA we applied (2mM) has been previously used by other laboratories [12,13,16]. Cheng et al used a higher concentration at 0.2%, while Soto-Guttierez et al preferred the use of EGTA, a similar compound that preferentially binds calcium ions [6,27]. Even though decellularization with EDTA-DET still retained approximately 10% of DNA, a slightly higher value when compared to published results demonstrating reduction to 0.5–5%, histological evidence demonstrates cell removal in both DET and EDTA-DET scaffolds [4–16,26,27].

Following demonstration of acellularity, we sought to assess the maintenance of the ECM components in both DET and EDTA-DET scaffolds. Histological examination using MT showed preservation of the portal and lobular connective tissue structure. PR, EVG, and AB staining demonstrated preservation of the collagen, elastin and GAG components respectively. Histological stains did not indicate any gross differences between the two scaffolds; however, we investigated the ECM components further using quantitative assays. Collagen was maintained to the same level as fresh tissue following DET treatment, as has been shown for SDS and TX100 protocols [4,11]. Interestingly, addition of EDTA increased the collagen content by 300%. The increase in collagen content compared with fresh tissue is probably due to normalization by wet weight (that includes the weight of cellular components in fresh tissue) coupled with the more compact nature of EDTA-DET scaffolds as shown by SEM, XPCI and vascular network analysis. We have previously demonstrated an increase in collagen following the decellularization process [17,26]. No protocols containing EDTA have quantified ECM components to compare with [6,12,13,27]. Similar results were seen with elastin quantification, with DET and EDTA-DET treatments reducing the elastin content to 30% and 40% respectively compared to fresh tissue, which is also comparable to the 10–50% reported in other published work [11,26]. GAG quantification is more complex since approximately 30% of GAG are associated with cell membranes [28,29], suggesting that any GAG loss is associated primarily with the process of decellularization and only secondarily with removal of GAG attached to ECM fibers. GAG are important for decellularized matrices as they are associated with factors that stimulate cell growth and differentiation that are essential for the successful transplantation of the seeded scaffolds. GAG quantifications demonstrated a reduction to around 20% of the fresh tissue values with EDTA having a slightly lower value. GAG in the EDTA-DET scaffold may be lower than that in DET due to the improved decellularization that removes more cellular membrane remnants and thus any associated GAG.

Since scaffold microarchitecture is dependent on the presence of collagen and elastin we investigated these proteins using immunohistochemistry and proteomics. Localization of specific ECM proteins (collagens I, III, IV, fibronectin and laminin) showed preservation following decellularization with no detectable differences between DET and EDTA-DET scaffolds. The collagens showed strong staining around the main blood vessels and weak parenchymal staining. Laminin staining was only positive around the major blood vessels, both in fresh and decellularized tissue, as reported previously [7,8,12,15]. Fibronectin staining was also consistent with the literature, appearing around blood vessels and within parenchyma [7,8,12]. Proteomics confirmed the presence of collagens, laminin, and fibronectin detected by IHC but not for collagen III. This may be due to low abundance, poor ionization or antibody specificity. Previous work that demonstrated the presence of growth factors following decellularization used semi-quantitative fluorometric assays [30]. No growth factors were detected using our shotgun mass spectrometric analysis due to the use of an SDS gel that limits the detection of low molecular weight proteins. Growth factors are also difficult to detect using a shotgun proteomic approach due to their low concentration. The preservation of laminins, fibronectin, nidogen-1 and type IV collagen suggests that the basement membrane of the decellularized scaffolds is maintained. However, the decrease in laminin and nidogen-1 levels in the EDTA-DET scaffolds suggests that addition of EDTA may partly degrade the basement membrane. A number of ECM proteins were not detected in fresh tissue. This is probably to a combination of reasons [31,32]: (i) ion suppression of peptides originating from ECM by peptides originating from the more abundant cellular components; (ii) incomplete in-gel digestion of ECM components in the presence of cellular proteins; (iii) limitations on the sensitivity and sequencing throughput of MS instruments; (iv) inadequate identification of peptides in MS/MS data; (v) insufficient pre-resolution of the peptides presented to MS/MS sequencing. It should be borne in mind that proteomic methods allow preferential identification of more abundant, easily digested proteins and ECM proteins provide a particular challenge for proteomics [33]. Our proteomics results are in keeping with recent work by Li et al who demonstrated uniquely identified ECM proteins in decellularized rat livers [34].

Electron microscopy and XPCI proved to be useful at revealing scaffold structure, as previously described in intestine and lung [17,18]. EDTA addition produced a denser scaffold with reduced space between the collagen fibers, which would account for the increased collagen and elastin levels in the tissue, since the matrix is more compact. Hepatic scaffolds with a denser structure have previously been produced, and even though these may retain ECM components, they may not be optimal for repopulation due to the collapse of cellular spaces [8,27,30]. The scaffold resulting from DET treatment retained the architecture of the portal tract as well as hepatocyte spaces surrounded by collagen fibers. This compares well to work performed by other groups who found that collagen fibers are weak and the general microarchitecture is poorly preserved after decellularization [9,13]. XPCI overcomes a basic limitation of conventional x-ray imaging, in which soft tissue contrast is lacking [35]. In this study, we used the simplest XPCI approach, free-space propagation [36] in its gold-standard synchrotron implementation, which has already been validated *in vivo* [37]. Later, translation to more compact, laboratory-based methods will be pursued [38].

The preservation of the vascular network is of paramount importance for liver tissue engineering, since a porous or destroyed ECM would result in the tissue being unacceptable for transplantation. This has previously been assessed by dye infusion though the PV/IVC [7,13,16,30], or by creation of vascular casts [4,6,9]. High concentrations of TX100 and SDS led to the destruction of the vascular network [9]. We have shown the preservation of the capillary network, which, interestingly, differed between DET and EDTA-DET scaffolds. Dye infusion was quantified and it was shown that dye intensity along with surface area coverage was increased in DET scaffolds. EDTA-DET scaffolds followed a two-stage increase in dye coverage. This may be due to the collapsed vascular spaces owing to the denser ECM. Following the build-up of sufficient pressure the distal vessels open up, allowing the passage of the dye.

We chose the DET scaffold as the optimal scaffold for cell seeding as although EDTA addition slightly increased the decellularization efficiency, it worsened architecture preservation as assessed by SEM, XPCI, and vascular network analyses. Previous cell seeding methodologies for the liver have focused on intravascular seeding of hepatic and/or endothelial cells [4,7,11,14,27]. Following seeding, cells were found to have organized around central vascular structures. However, movement across the blood vessel walls would require extravasation across the basement membrane. The mechanisms suggested for such cell migration include proteolytic digestion, mechanical force and variability in basement membrane composition [39]. In scaffolds where there are no cells to repair the ECM following proteolytic and/or mechanical degradation, the blood vessels may become perforated and leaky, preventing its use for transplantation. We sought to avoid this by performing seeding using multifocal microinjection, allowing wide and homogeneous cell distribution, engraftment and survival in a three-dimensional matrix culture condition. Cells were alive and proliferating 2 weeks following *in vitro* seeding, completely repopulating the scaffold, similarly to published results with intravascular cell infusion [4,7,11,14,27]. The scaffold’s architecture and composition enabled cell seeding and adhesion that, coupled with oxygen and nutrient exchange, allowed cellular expansion and scaffold repopulation. We aim to expand on this by performing concomitant intravascular seeding of endothelial cells.

## Conclusions

The present work demonstrates the production of a natural acellular matrix that can be obtained from rat liver by DET decellularization while preserving microarchitecture and ECM components. Addition of EDTA creates a denser, compact matrix, and decreases residual DNA content. The DET scaffold is biocompatible, allowing cell adhesion and growth for up to 14 days. The fine-tuning of the protocol including scaling up of the methodology, optimization of cellular reseeding and transplantation studies are critical for the long-term goal of providing a potential therapeutic alternative to conventional transplantation.

## Supporting Information

S1 Table Proteomics full data table. Master table of MS data summarizing all the proteins identified in each sample (Fresh, DET, and EDTA-DET).
(XLSX)

S1 Video Synchrotron-based XPCI of DET scaffold. (DIVX)

S2 Video Synchrotron-based XPCI of EDTA-DET scaffold. (DIVX)

S3 Video Vascular network imaging of DET scaffold. (AVI)

S4 Video Vascular network imaging of EDTA-DET scaffold. (AVI)
